# Supplementary material for: Ribonuclease activity undermines immune sensing of naked extracellular RNA
Source: bioRxiv. 2024 Apr 23:2024.04.23.590771. Preprint. [Version 1] doi: 10.1101/2024.04.23.590771 (PMC11071435; doi:10.1101/2024.04.23.590771)
Supplement: Supplement 1 [file media-1.pdf]

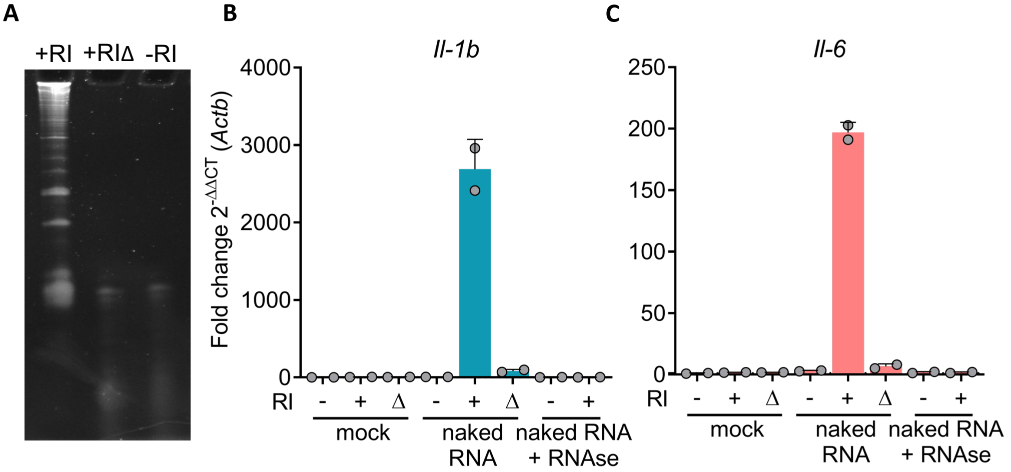

Supplementary Figure 1. Inflammatory naked extracellular RNA recognition is completely dependent on RI enzymatic activity.

A) PAGE under denaturing conditions of U-2 OS total RNA incubated in 10% fetal bovine serum together with 400 U / mL RI (+RI) or heat inactivated RI (+RIΔ) or without RI (-RI) for 45 minutes. **B,C)** *Il-1b* **(B)** and *il-6* **(C)** expression by RT-qPCR in BMDCs stimulated for 6 hs with 1 µg / mL naked total RNA from *E. coli* with RI 80 U / mL (+RI) or heat inactivated RI (+RIΔ) or without RI (-). RNAse1 treated *E. coli* RNA with (+RI) or without RI (-) were used as control and DPBS as negative control.

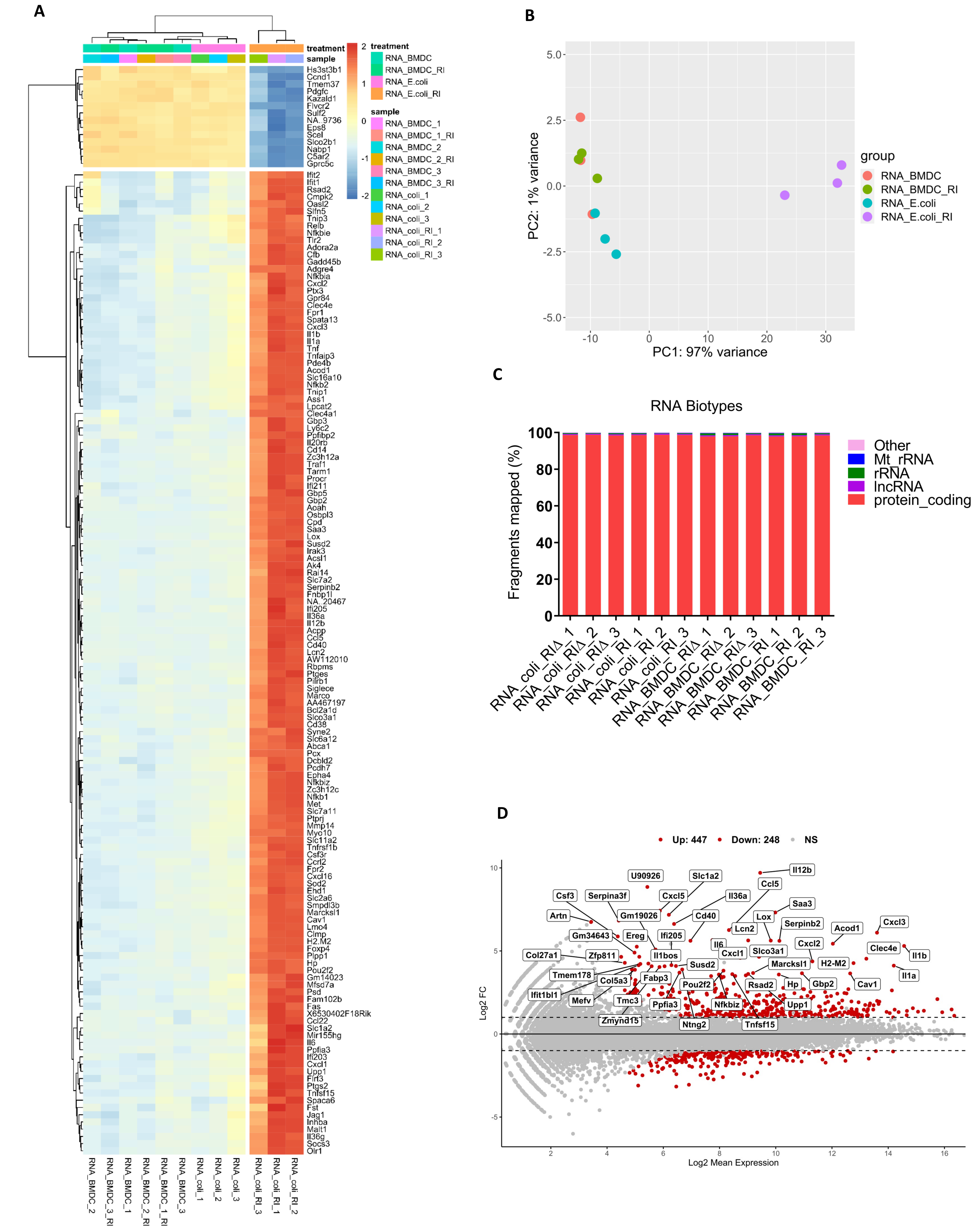

Supplementary Figure 2

A) Heatmap showing top 150 most differentially regulated genes in BMDCs stimulated for 6hs with 100ng/ml naked total RNA from *E. coli* or 100ng/ml total RNA from BMDCs in the presence of RI 80 U/ml (+RI) or thermally inactivated RI (+RIΔ). **B)** PCA plots corresponding to all 12 experimental conditions. **C)** RNA biotypes abundance across samples. **D)** MA-plot between BMDCs stimulated with 100ng/ml naked total RNA with RIΔ or RI.

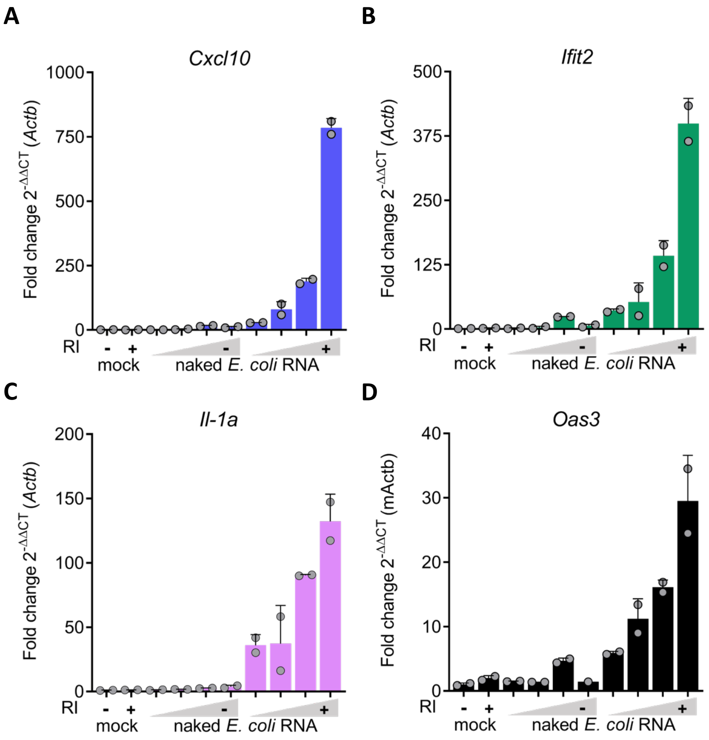

Supplementary Figure 3

Cxcl10 **(A)** *ifit2* **(B)** *Il-1a* **(C)** *and Oas3* **(D)** expression by RT-qPCR in BMDCs stimulated for 6hs with varying doses (1; 5; 10; 25 µg / mL) of naked total RNA with or without RI 80 U / mL. DPBS was used as negative control.

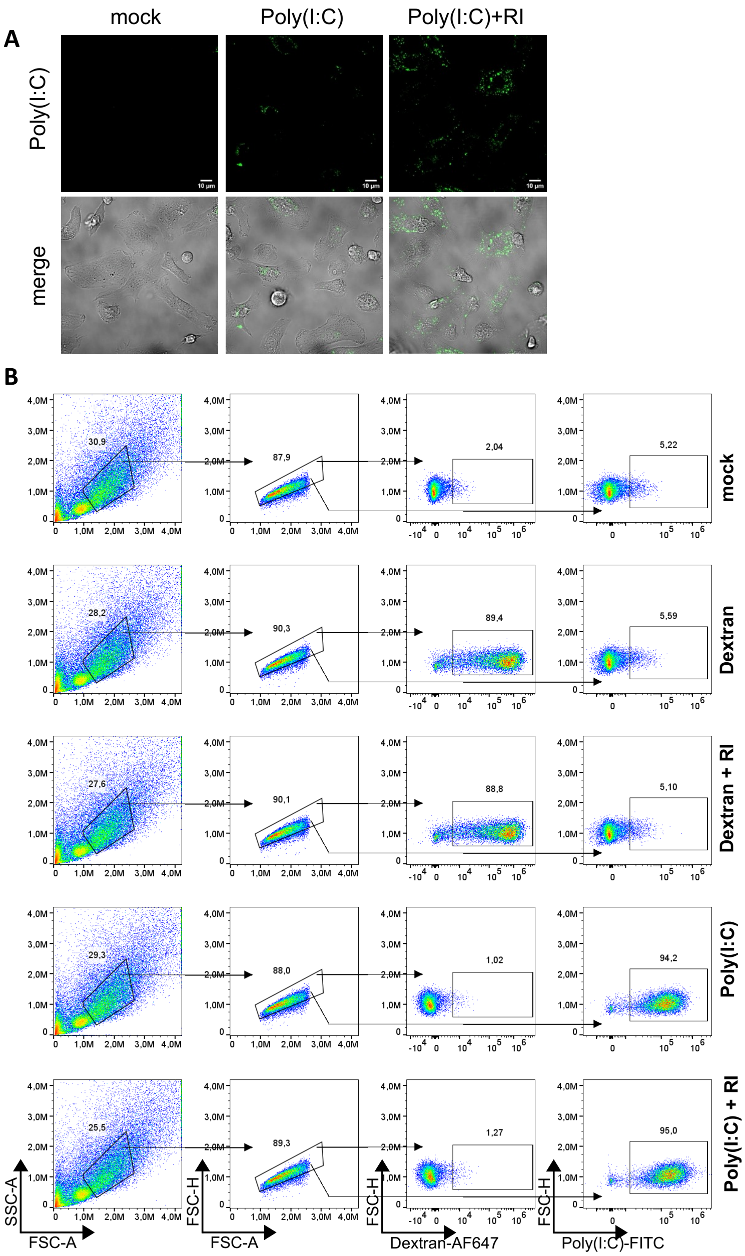

Supplementary Figure 4. Internalization of naked fluorescent RNA by BMDCs

A) Confocal microscopy of BMDCs cultured in 10% FBS left untreated or stimulated with naked Poly(I:C)-Fluorescein 0.5 μg / mL with or without RI for 1 hr. **B)** Flow cytometry of BMDC cultured in 10% FBS and stimulated for 1 hr with naked Poly(I:C)-Fluorescein 0.5 μg / mL or Dextran-AF647 with or without RI 120 U / mL.

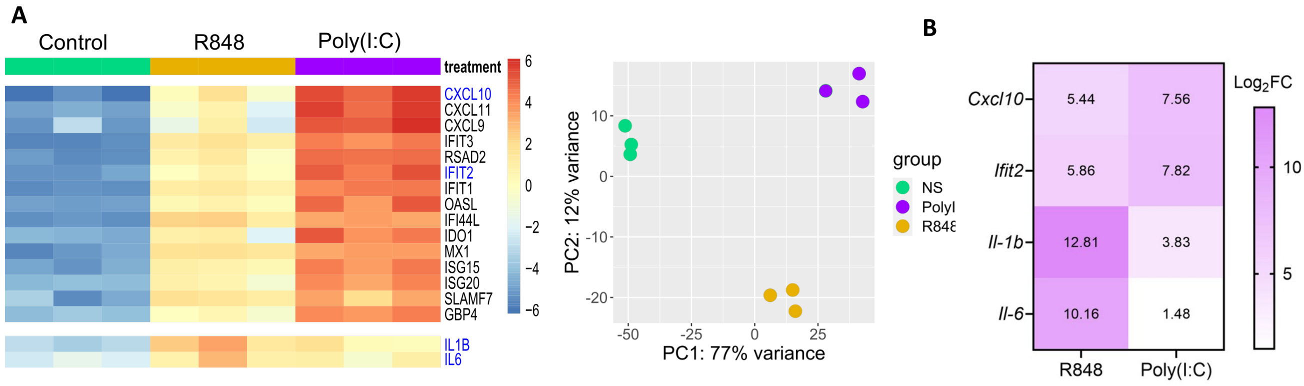

Supplementary Figure 5

A) Data mining and differential gene expression analysis of a transcriptomic dataset (PMID:31968263, GEO:GSE125817) from monocyte derived dendritic cells stimulated with Poly(I:C) or R848 for 6 h. A heatmap with the top 15 most upregulated genes (including *Il-1b* and *Il-6*) and the corresponding PCA plot are shown. **B)** Heatmap showing *Cxcl10, Ifit2, Il-1b* and *il-6* expression by RT-qPCR in BMDCs stimulated for 6hs with 100 ng / mL R848 or 10 µg / mL poly(I:C). DPBS was used as negative control.

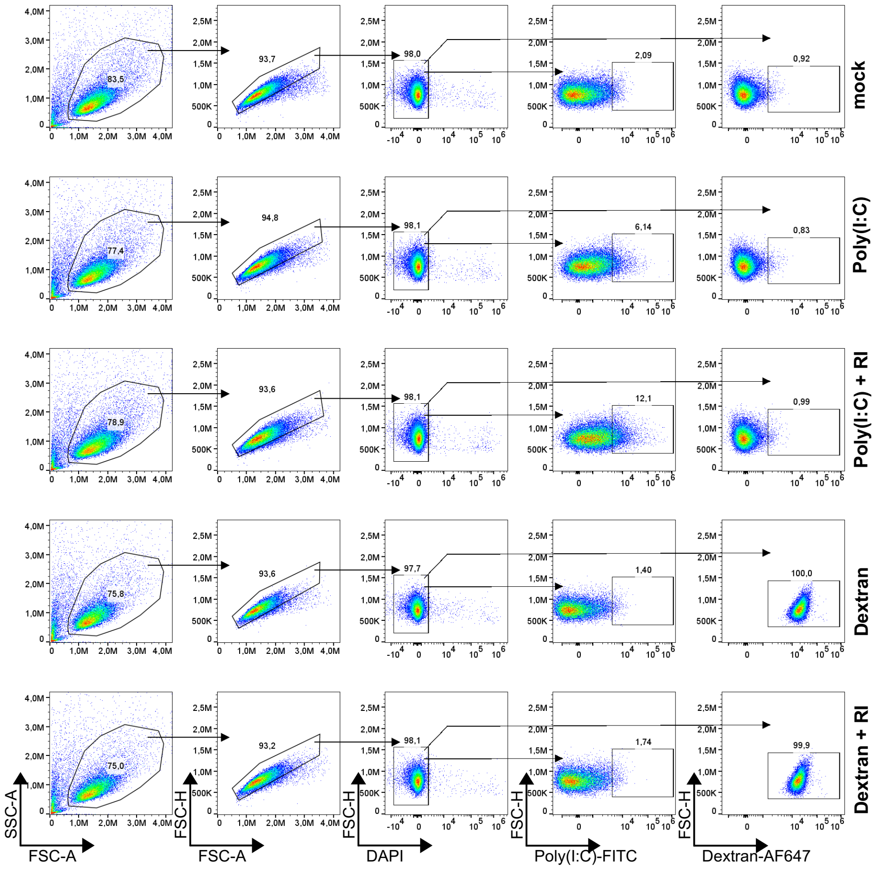

Supplementary Figure 6. Internalization of naked fluorescent RNA by THP1 cells

Flow cytometry of THP1 cells cultured in 10% FBS and stimulated for 1 h with naked Poly(I:C)-Fluorescein 0.5 µg / mL or Dextran-AF647 with or without RI 120 U / mL.

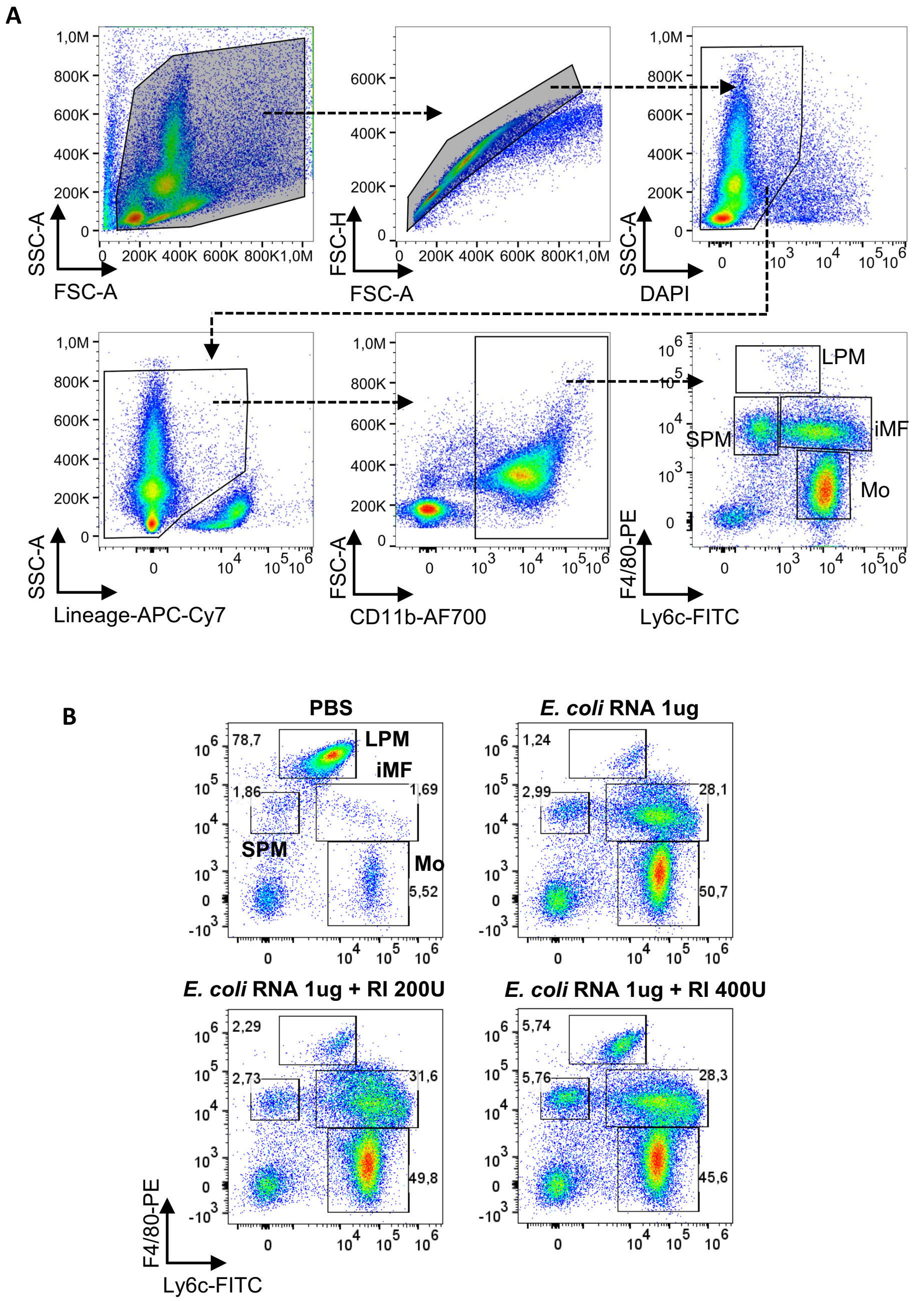

Supplementary Figure 7. Gating strategy to study of mice peritoneal cells

Flow cytometry gating strategy to analyze resident large (LPM) and small (SPM) peritoneal macrophages, infiltrating monocytes (Mo) and inflammatory macrophages (iMF). Gating strategy shows cells 24 h after *i.p* administration of 12,5 µg of total naked *E. coli* RNA **(A)** Peritoneal cell populations gated under CD11b+Lineage- 24hs after administration of PBS or naked *E. coli* RNA 1 µg with the indicated RI dose **(B).** Percentages correspond to the parental gates.

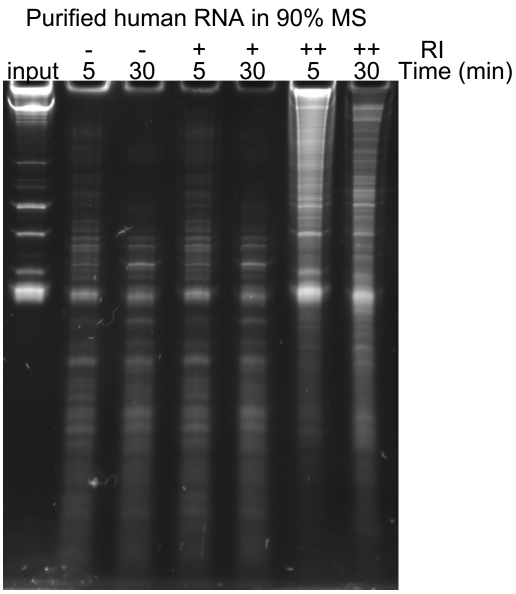

Supplementary Figure 8.

PAGE under denaturing conditions of human total RNA incubated in 90% mouse serum for either 5 or 30 minutes in the presence of 160(++), 40(+) U / mL of RI or no RI(-).

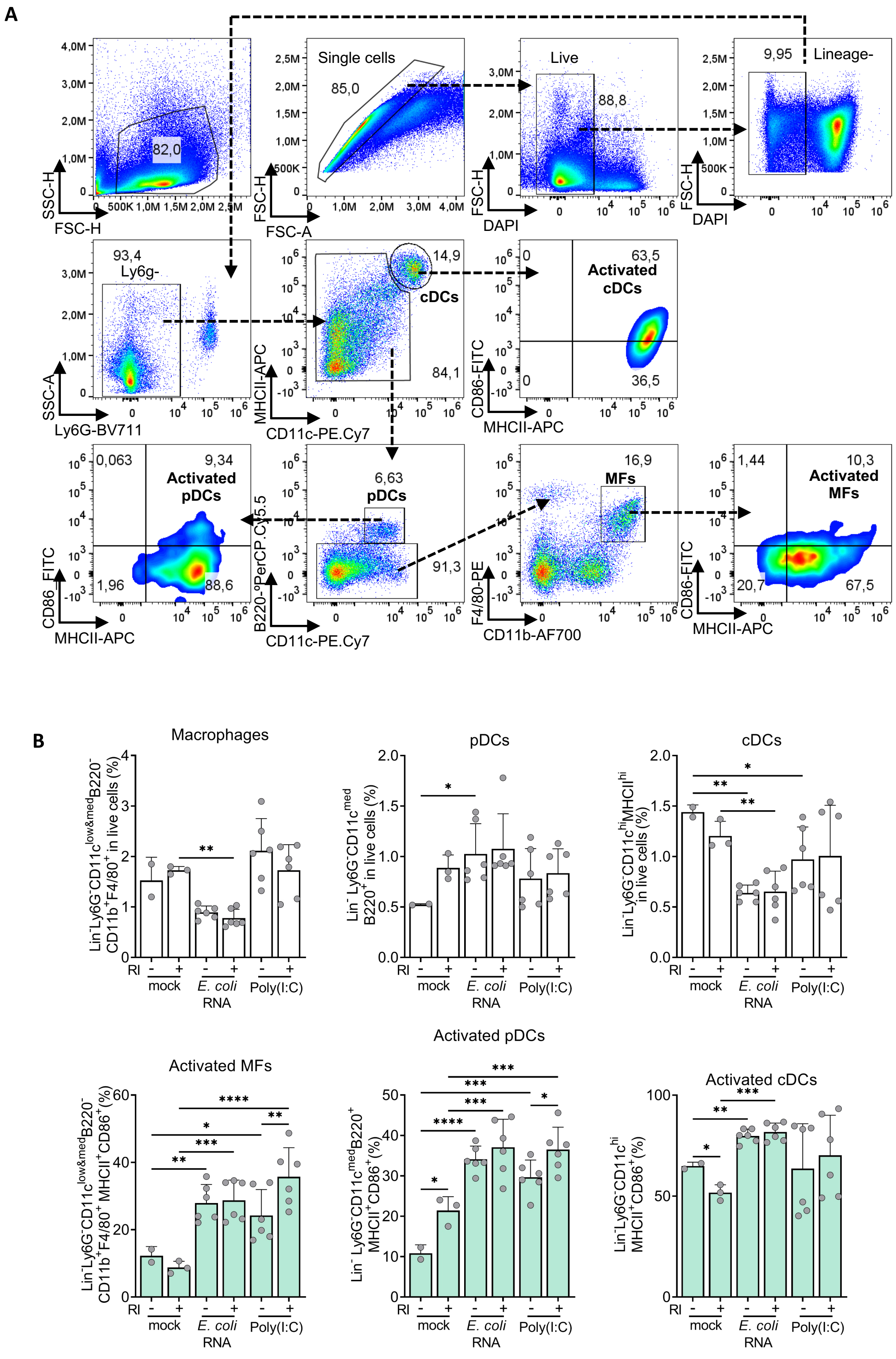

Supplementary Figure 9. Effect of naked exRNA in vivo on splenic myeloid cells and gating strategy

A) Flow cytometry gating strategy to study splenic cDC, pDC and macrophages after intravenous administration of naked RNA stimuli. Percentages correspond to the parental gates.**B)** Flow cytometry analysis of spleen cells 6 h after intravenous administration of either naked Poly(I:C) 200ng, naked total *E. coli* RNA 10 µg with or without 480 units of RI. PBS was used as mock. The percentage of total Macrophages (Lin$^-$Ly6G$^-$CD11c$^{low\&med}$B220$^-$CD11b$^+$F4/80$^+$), pDCs (Lin$^-$ Ly6G$^-$CD11c^{med}B220$^+$), cDCs (Lin$^-$Ly6G$^-$CD11c^{hi}MHCII^{hi}), gated in live cells is shown in top panels. The percentage of activated macrophages, pDCs, cDCs, (defined as MHCII$^+$CD86$^+$) gated under their corresponding cell type is shown in bottom panels with bar plots shaded in green.

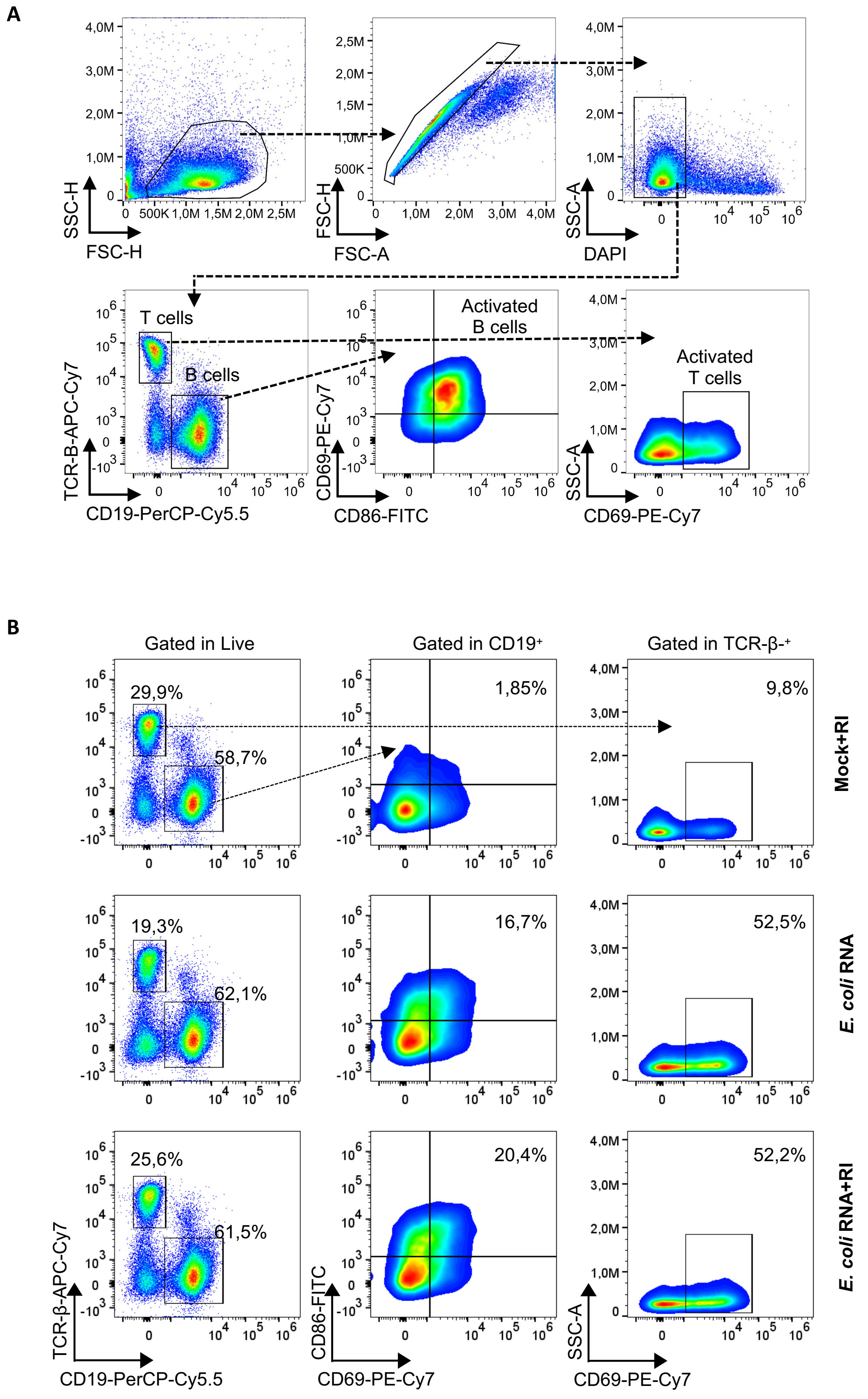

Supplementary Figure 10. Effect of naked exRNA in vivo on splenic lymphoid cells and gating strategy

A) Flow cytometry gating strategy to study splenic B and T cells populations after intravenous administration of RNA stimuli. Percentages correspond to the parental gates. **B)** Dot plots showing flow cytometry analysis of spleen cells 6 h after intravenous administration of naked total *E. coli* RNA 10 µg with or without 480 units of RI and PBS with RI. Percentages correspond to the parental gates.
