# Supplementary material for: Ribonuclease activity undermines immune sensing of naked extracellular RNA
Source: bioRxiv. 2024 Apr 23:2024.04.23.590771. Preprint. [Version 1] doi: 10.1101/2024.04.23.590771 (PMC11071435; doi:10.1101/2024.04.23.590771)
Supplement: Supplement 2 [file NIHPP2024.04.23.590771v1-supplement-2.pdf]

bioRxiv preprint doi: https://doi.org/10.1101/2024.04.23.590771; this version posted April 23, 2024. The copyright holder for this preprint (which was not certified by peer review) is the author/funder, who has granted bioRxiv a license to display the preprint in perpetuity. It is made available under aCC-BY 4.0 International license.

SUPPLEMENTARY FIGURES

See Supplementary Figures file for Fig S1 – S10

SUPPLEMENTARY TABLES

Table S1. Primers for RT-qPCR

Gene	Forward Primer	Reverse Primer
murine *Il6*	5'-AAGCCAGAGTCCTTCAGAGAG-3'	5'-GTCTTGGTCCTTAGCCACTCC-3'
murine *Il1a*	5'-AGTCAACTCATTGGCGCTTG-3'	5'-AGAGAGAGATGGTCAATGGCAG-3'
murine *Cxcl10*	5'-AGTGCTGCCGTCATTTTCTG-3'	5'-GCAGGGATGATTTCAAGCTTCC-3'
murine *Ifit2*	5'-TGCTTTGAGCGCTTTGACAC-3'	5'-TCGCAGATTGCTCTCCAGTG-3'
murine *Oas3*	5'-AGGCTACCGTGTACGCATC-3'	5'-CTTCACACAGCGGCCTTTACC-3'
murine *Il1b*	5'-TGCCACCTTTTGACAGTGATG-3'	5'-ATGTGCTGCTGCGAGATTTG-3'
murine *Actb*	5'-TGGCTCCTAGCACCATGAAG-3'	5'-AACGCAGCTCAGTAACAGTCC-3'
NanoLuc	5'-ATGGTCTTCACACTCGAAGATTTCG-3'	5'-CTGGACACACCTCCCTGTTC-3'

Table S2. Primers for IVT template amplification

Gene	Forward Primer	Reverse Primer
NanoLuc	5'-ACGACGTAATACGACTCACTATAGG<br>GTATCCGCCACCATGGTCTTCACACTCG-3'	5'-TTTTTTTTTTTTTTTTTTTTTTTTTTTTTT<br>CGACTCTAGAATTATTACGCCAGAATG-3'
eGFP	5'-ACGACGTAATACGACTCACTAT<br>AGGGTATCCGCCACCATGGTGAGC-3'	5'-TTTTTTTTTTTTTTTTTTTTTTTTTTTTTTT<br>ACTTGTACAGCTCGTCCATGC-3'

Table S3. Antibodies mixes employed

bioRxiv preprint doi: https://doi.org/10.1101/2024.04.23.590771; this version posted April 23, 2024. The copyright holder for this preprint (which was not certified by peer review) is the author/funder, who has granted bioRxiv a license to display the preprint in perpetuity. It is made available under aCC-BY 4.0 International license.

.	Antibodies	Concentration
BMDCs	CD40-FITC	1/500
	CD86-PE	1/200
	MHCII-APC	1/200
Spleen myeloid cells	CD19-APC-Cy7	1/200
	TCR-B-APC-Cy7	1/200
	Ly6G-BV711	1/200
	CD11c PE-Cy7	1/200
	MHC-II-APC	1/200
	B220-PerCP-Cy5.5	1/200
	F4/80-PE	1/200
	CD11b-AF700	1/200
	CD86-FITC	1/500
Spleen lymphoid cells	CD19-PerCP-Cy5.5	1/200
	TCR-B-APC-Cy7	1/200
	CD69-PECy7	1/200
	CD86-FITC	1/500
Peritoneal macrophages and monocytes	CD19-APC-Cy7	1/200
	TCR-B-APC-Cy7	1/200
	CD11b-AF700	1/200
	F4/80-PE	1/200
	Ly6c-FITC	1/500
